# Homœopathy

**Published:** 1856-12

**Authors:** Robert E. Campbell

**Affiliations:** Benton, Lowndes County, Alabama


A T L A N T A

### Medical and Surgical Journal.

Vol. II.] DECEMBER, 1856. [No. 4.

### ORIGINAL COMMUNICATIONS.

### ARTICLE I.

*Homœopathy.* By Robert E. Campbell, M. D., Benton, Lowndes County, Alabama.

Quackery is one of the social evils; indeed, in a free government, it is an inalienable right. Attack Quacks and they will turn it to their pecuniary interests—they will cry out *persecution.* Homœopathy is the most *decent* if not the most *rational* system of quackery now extant, and if it were carried out it would fulfil one of the golden rules of Therapeutics—*do no harm;* or, as Moses hath it, "*Thou shalt not kill*"—though in a violent disease requiring active medication, infinitesimals would surely *do no good.* Homœpathy will have its day, and it ought to be encouraged in the hope that it may displace many worse systems. It was Burke who said of the beautiful: "it must be small without angles;" (Query:—Did he refer to Homœopathic globules?)

In the following pages we wish it to be clearly understood that it is not our purpose to convince Homœopathic doctors, male or female, of their errors. We confess ourselves unequal to such a task, and as Homœopathy cannot be brought down to *terra firma*, we must permit these animated cabbages to march unrestricted over their gossamer parade ground. Controversies which have their seat beyond the limits of known existence, are not to be decided by human instrumentality.

We will not weary our readers with unnecessary refutation of Homœopathic dogmas. They are self-evident absurdities, and we might as well occupy time

> "——to prove by force
> Of argument that man's no horse,"

as to show their folly. Every one of them is positively contradicted, both by reason and fact, and none of them are sustained by one-hundredth part of the evidence which proclaims Joe Smith a prophet.

But the practical absurdity of Homœopathy, in the matter of doses, is worth some consideration, because to some of our readers it may be novel, and because, to us all, it forms an illustration of human credulity, perhaps more forcible than any other which can be presented from the modern history of man.

A ray of reason seems to have flashed across the mind of Habnemann, when he resolved that a *theory*, in which there was not a particle of sense, should beget a *practice* in which there was not a particle of physic. It may possibly have occurred to him that men who could admit his dogmas, were not in a proper condition of mind to be trusted with drugs. Be this as it may, he promulged the extraordinary doctrine that *the potency of medicine is in proportion to the exiguity of the dose; and that diseases are only successfully combated by infinitesimal atoms of medicine, which possess the power to induce in healthy persons diseases similar to those for which they are antidotes;* the latter proposition serving the purpose of obviating the natural objection to the first, drawn from every-day observation of the power of drugs, inasmuch as it removed the ground of observation from observable phenomena to conjectural effects.

Nothing can be more probable than that Hahnemann was himself a skeptic in the powers of medicine, and considered his system what it really is, an abandonment of diseases to the unaided curative efforts of nature. The queer theory which he invented must be regarded in the light of a myth, in which a supposed truth not likely to be received by the vulgar, is so wrapped up in acceptable obscurity and pompous foolery, as to gain practical currency.

Few of our readers, probably, have any idea of the insane extent to which the doctrine of multiplying the power by dividing the dose of medicine, is carried by the Homœopaths.

The patients of these pretenders, naturally influenced by old associations, look upon the little pillets, solemnly administered to them, with the awe which the presence of power inspires. Their imagination is wonderfully strict, by the Lilliputian embodiment of Titanic forces, and they put forth the trembling hand to receive the potent pillule with much the same feeling with which one handles percussion powder, or the hair-trigger of a rifle. It is impossible to persuade these people that they are imposed upon by their old habits of thought, and that homœopathic pharmacy is not a refined process of concentration, but a gross method of dilution. Many persons refuse to believe the statements made, with regard to the mode of preparation of homœopathic doses; but upon this subject there can be no doubt or dispute. Homœopaths openly avow, and publish their plan of pharmacy, funny as it is, and the uncheated community may fully enjoy the development without any misgiving as to their honest title to mirth.

The homœopaths assert that, "in order to obtain the full power of a drug, it must be reduced to an *infinitesimal* atom." Now, an "infinitesimal" is necessarily an indefinite and indefinable thing; it passes the power of conception; it is less than any assignable quantity. In preparing such a dose, a difficulty, if not impossibility, must be overcome. We must be satisfied with an approximation to it; but this approximation Hahnemann has cleverly achieved. In order to prepare a pillule, a grain of any ordinary substance, sulphur, silex or mercury, is rubbed up with ninety-nine grains of sugar of milk. Out of this mass a single grain is taken, and subjected to a second admixture, and so on to the requisite dilution. If the substance to be diluted be a fluid instead of a grain, a drop of any of our ordinary tinctures is taken, and mixed with ninety-nine drops of water—from this a drop is taken and mixed with another ninety-nine drops, and so on. The thirtieth dilution is frequently employed; and to administer their doses, pills of the sugar of milk are moistened with a drop of the ultimate preparation and there *dried!!!* The patient may, according to directions, *swallow*, or *smell* their *doses*. By this ingenious process, an ordinary drug, which, as a whole grain, has no visible power, according to homœopathy, becomes a tremendous potency to the "immaterial principle."

Hahnemann says, that the most appropriate dose is the thirtieth dilution—which consists of a decillionth of a grain or drop. Dr. Simpson endeavors to illustrate the subject thus: "There are, upon the earth, some 900,000,000 human beings. If all these had been called into being when Adam was created, some 6,000 years ago, and had lived up to the present time, and each of these 900,000,000 individuals had, when first called into existence, began to swallow, and continued to swallow up to the present hour, without rest or cessation, night and day, a decillionth dose of a grain, they would not yet have finished a single grain of the medicine. Nay, if each of these 900,000,000 men, now 6,000 years old, had swallowed, during every moment of their past existence, not a single globule but *one million* of globules of Hahnemann's thirtieth dilution, they would not yet have finished a single grain, and would not finish it, working constantly, at the same rate, for millions of centuries to come."

A great number of similar illustrations might be quoted, but the above will be enough to give our readers as much information in homœopathic mensuration and arithmetic, as their minds will be capable of receiving at once. We will now barely hint to them, that after mastering the profound nothingness of the thirtieth dilution, they may extend their calculations to the six thousandth, which, we are gravely assured, is good for consumption. To obtain an idea of this infinitesmality, we may suppose it to represent the amount of common sense to be extracted from a universal space full of homœopaths, or the quantum of modesty, resident in Hahnemann. It is a flight of folly, which far distances common arithmetic, and leaves, exhausted logarithms panting in the rear.

It is certain that homœopaths give no medicine at all. If matter be infinitely invisible, it cannot be infinitely divided. To effect this there must not only be susceptibility of division, but instruments to effect it. Grains of sugar may penetrate and divide bodies of certain bulk, there is no reason to believe they can do this to bodies infinitely small, nor that globules of water can continue *ad infinitum* to dissolve other fluids. A pestle and mortar would be gross instruments wherewith to operate upon millionth and billionth particles. Compared with such exiguities, the fissures in our smoothest mortars would be mammoth caves. But the whole thing is supremely absurd and needs no refutation.

Yet, how comes it, say the homœopaths, that our little pills cannot be swallowed with impunity, as has been frequently proven to the sorrow of sufferers, who have bolted a hundred of them in derision? It is well known that many persons practicing homœopathy, fill their vials with concentrated medicines. Arsenic, morphine, aconite, &c., are their ordinary prescriptions. Such men are simply *scoundrels.* They are sacrificing both health and life, under the pretence of administering what ought to be innocent pilloids. They are not homœopaths in theory or practice, but living on false pretences.

It is somewhat strange how their numerous Apothecary shops and pharmacies are supported. Certainly, the sale of homœopathic physic could not furnish occupation for one Apothecary to the world, for a single grain of medicine would be sufficient to medicate mankind through all eternity.

The queer conceit that medicines are specifically curative of diseases, the like of which they generate, is worthy of some notice, as another instance of the power of dogmatism to control the understanding. The assumption is without any support from fact, much less based upon ordinary observation. Every one is aware, that in many instances, opium lulls pain, but no one administers it because it produces pain. Castor oil excites the sluggish peristaltic action of the alimentary canal, yet, does it follow that, upon the healthy subject, it acts as an astringent? Ipecacuanna vomits the sick, and, also the well. Many more instances might here be adduced, but the foregoing will suffice to show the absurdity.

In the instances of "*similia similibus curantur*," adduced by Hahnemann, are startling exhibitions of the superficial quality of his mind, and of his natural proneness to sophistical conclusions. He formed, it seems, his exposition of medicinal agencies from the proverb, "a hair of the dog that bit you;" but he has failed to comprehend the philosophy of it. And, upon the mere *ipse dixet* of this imprudent man, we are asked to destroy all that the human mind has accumulated concerning the power of drugs; to surrender our splendid *materia medica*, with all its oft-tried stores; to abandon all the results of observation on the nature, progress, and treatment of disease, and to trust for relief from suffering and premature death, to infinitesimal dilutions. Great numbers of persons, called educated, comply with this demand. Even the Czar of Russia permitted himself to be made a fool of by a homœopath—doubtless, never having given the subject a moment's consideration. The Emperor of all the Russias was permitted to die with as little scientific aid as an Australian savage.

[TO BE CONTINUED.]

